# Quantitative analysis of intermolecular interactions in orthorhombic rubrene

**DOI:** 10.1107/S2052252515012130

**Published:** 2015-08-14

**Authors:** Venkatesha R. Hathwar, Mattia Sist, Mads R. V. Jørgensen, Aref H. Mamakhel, Xiaoping Wang, Christina M. Hoffmann, Kunihisa Sugimoto, Jacob Overgaard, Bo Brummerstedt Iversen

**Affiliations:** a Center for Materials Crystallography, Department of Chemistry and iNANO, Aarhus University, Langelandsgade 140, Aarhus C DK-8000, Denmark; b Chemical and Engineering Materials Division, Neutron Sciences Directorate, Oak Ridge National Laboratory, PO Box 2008 - MS 6475, Oak Ridge, TN 37831, USA; c Japan Synchrotron Radiation Research Institute, I-I-I, Kouto, Sayo-cho, Sayo-gun, Hyogo 679-5198, Japan

**Keywords:** electron density, rubrene, organic semiconductor, interaction energy

## Abstract

Rubrene is one of the most studied organic semiconductors to date due to its high charge carrier mobility which makes it a potentially applicable compound in modern electronic devices. Previous electronic device characterizations and first principles theoretical calculations assigned the semiconducting properties of rubrene to the presence of a large overlap of the extended π-conjugated core between molecules. We present here the electron density distribution in rubrene at 20 K and at 100 K obtained using a combination of high-resolution X-ray and neutron diffraction data. The topology of the electron density and energies of intermolecular interactions are studied quantitatively. Specifically, the presence of C_π_⋯C_π_ interactions between neighbouring tetracene backbones of the rubrene molecules is experimentally confirmed from a topological analysis of the electron density, Non-Covalent Interaction (NCI) analysis and the calculated interaction energy of molecular dimers. A significant contribution to the lattice energy of the crystal is provided by H—H interactions. The electron density features of H—H bonding, and the interaction energy of molecular dimers connected by H—H interaction clearly demonstrate an importance of these weak interactions in the stabilization of the crystal structure. The quantitative nature of the intermolecular interactions is virtually unchanged between 20 K and 100 K suggesting that any changes in carrier transport at these low temperatures would have a different origin. The obtained experimental results are further supported by theoretical calculations.

## Abstract 2

A combination of single-crystal X-ray and neutron diffraction experiments are used to determine the electron density distribution in orthorhombic rubrene. The topology of electron density, NCI analysis and energetics of intermolecular interactions clearly demonstrate the presence of π⋯π stacking interactions in the crystalline state.

## 1. Introduction   

Semiconductors are essential components of all modern electronic devices that we depend on in our daily life. Recently, organic semiconducting materials based on acenes, heteroacenes and thiophenes have received an intensive academic and commercial interest due to their promising optoelectronic and charge transfer properties (Coropceanu *et al.*, 2007 ▸; Murphy & Fréchet, 2007 ▸; Yassar, 2014 ▸). The design and synthesis of new functional π-conjugated materials is a major interest in the scientific community aiming to develop all organic or hybrid organic inorganic electronic devices such as Organic Light Emitting Diodes (OLED) (Reineke *et al.*, 2013 ▸), Organic Field Effect Transistors (OFET) (Facchetti, 2007 ▸; Reese & Bao, 2007 ▸; Yamashita, 2009 ▸; Dong *et al.*, 2010 ▸), photovoltaic cells (Mishra & Bäuerle, 2012 ▸), sensors (Mannsfeld *et al.*, 2010 ▸) and radio frequency identification (RFID) tags (Subramanian *et al.*, 2005 ▸). Field effect hole and electron mobilities as high as 40 cm^2^ V^−1^ S^−1^ and 11 cm^2^ V^−1^ S^−1^ respectively, have been achieved for organic semiconductors which, remarkably, are higher than those seen for amorphous silicon (Jurchescu *et al.*, 2007 ▸; Li *et al.*, 2012 ▸). The main advantage of using organic semiconducting materials in device fabrication is that they offer a low cost alternative to silicon; they are also light-weight, and their synthesis and easy processing into devices makes them extremely flexible. Indeed, they can be suitably modified to meet the compatibility of solution process techniques in contrast to expensive lithography and vacuum deposition methods, normally needed for their inorganic counterparts. However, the performance of most recent organic electronic devices is still limited by the relatively low carrier mobility compared with inorganic materials.

To achieve higher mobility of charge carriers in organic materials, the molecule must possess an extended π-conjugated core and it must crystalize with strong π⋯π overlap between the molecules in the crystalline state (Yassar, 2014 ▸). It is often assumed that crystal packing of molecules in herringbone, one-dimensional or two-dimensional planar π-stacking motifs with the absence of edge-to-face interactions would result in higher charge mobility. Edge-to-face interactions result in a slippage of aromatic stacking and thus, lower charge mobility. However, a quantitative correlation of the microscopic molecular and crystal structure properties with the macroscopic properties such as the mobility is still lacking in organic semiconductors.

**Figure 1:**
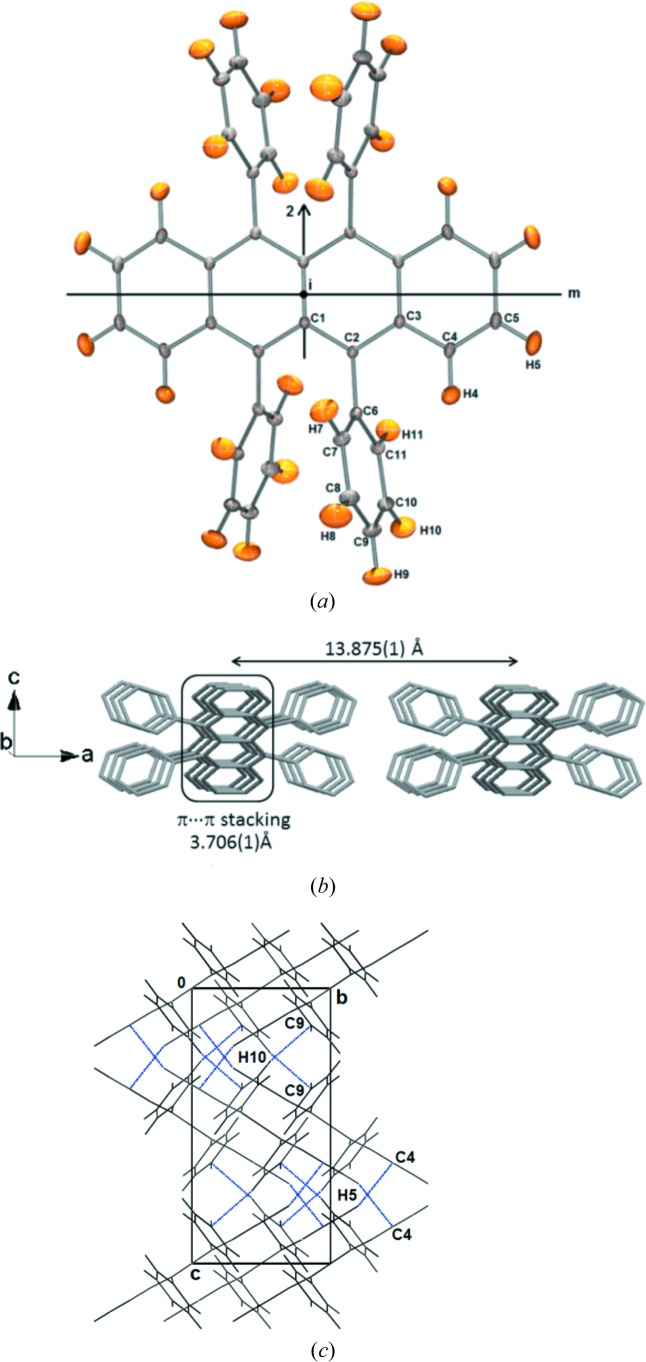
(*a*) *ORTEP* representation of the structure of rubrene at 100 K. Atom ellipsoids are shown at 50% probability level. The symmetry operations 2, *m* and *i* are superimposed on the molecule. (*b*) Molecular packing diagram with π⋯π stacking along the *b* axis and interplanar distance between layers along the *a*-axis at 100 K. (*c*) Herringbone packing of molecules depicting C4⋯H5 and C9⋯H10 interactions (blue dotted lines), viewed along the *a* axis.

Single crystals of organic semiconductors have been the main focus of research as they provide relevant information about intrinsic and anisotropic charge mobilities and they have several advantages over polycrystalline thin films (Podzorov, 2013 ▸; Jiang & Kloc, 2013 ▸; Lezama & Morpurgo, 2013 ▸). The primary advantage is the availability of high purity compounds for device fabrication and minimization of the charge trapping by elimination of grain boundaries in single crystals. On the other hand, growing large single crystals for device fabrication is time consuming and the resulting crystals are often very brittle. Additionally, it is difficult to make electrical contacts without introducing any strain or damage to the crystals (Podzorov, 2013 ▸). The orthorhombic polymorph of rubrene (5,6,11,12-tetraphenyltetracene) is one of the most explored organic semiconductors due to its attractive semiconducting properties. It has nearly 100% fluorescence quantum efficiency in solution (Strickler & Berg, 1962 ▸), which is promising for the design of OLEDs. Single crystals show *p*-type characteristics with high charge mobility up to 20 cm^2^ V^−1^ S^−1^ (Podzorov *et al.*, 2004 ▸; Hulea *et al.*, 2006 ▸; Hasegawa & Takeya, 2009 ▸; Takahashi *et al.*, 2006 ▸). The large charge-carrier mobility measured in rubrene has been attributed to the herringbone packing motif in the orthorhombic crystalline polymorph which provides high spatial overlap of the π-conjugated tetracene backbone (see Fig. 1 ▸ *c*). The charge transport properties of rubrene are severely affected when it undergoes an oxidation in the presence of UV light and O_2_ to form rubrene endoperoxide, which lead to less aromaticity in the tetracene backbone by a formation of a kink in the backbone and consequently a loss of the π-stacking interactions (Fumagalli *et al.*, 2011 ▸; Mastrogiovanni *et al.*, 2014 ▸) as well as the semiconducting properties. Recently, the mechanical properties of rubrene single crystals have also been investigated to understand the performance limit, processability and design of devices using single crystals (Reyes-Martinez *et al.*, 2012 ▸). A relationship between the mechanical properties and the crystal structure of rubrene was derived by determining the in-plane elastic constants in the [001] and [010] directions. Interestingly, a peak value for the buckling wavelength was experimentally observed at approximately 30° with respect to the [010] direction which corresponds to the angle between the tetracene backbone stacking and the *b* axis in the crystal packing. The monoclinic and triclinic polymorphs of rubrene show poor semiconducting properties due to the absence of π-stacking interactions in the monoclinic polymorph and significant slippage of tetracene backbones in the crystal packing of the triclinic polymorph. Recent calculations of the transfer integral (*t*) and the band structure have been used to study the semiconducting properties of rubrene (McGarry *et al.*, 2013 ▸). In rubrene, the largest *t* values of 100 meV and 53 meV were obtained for holes and electrons, respectively, along the π-stacking direction corresponding to the *b* axis. Further, full periodic electronic band structure calculations in the crystal geometry suggest that the top of the valence band and bottom of the conduction band are found at the Γ-point, indicating a direct band gap for rubrene. In the band structure calculations, the Γ–Y direction in the Brillouin zone corresponds to the π–π coupling in the *b* direction of the unit cell and the herringbone packing motif in the *c* direction of the unit cell was represented by the Z point in the Brillouin zone (McGarry *et al.*, 2013 ▸). It was shown that the largest *t* values and π–π coupling along the *b* axis is due to the presence of π⋯π intermolecular interactions in the rubrene molecules.

To gain further insight into intermolecular interactions which control the transport properties, we have studied the electron density (ED) distribution in orthorhombic rubrene using high-resolution low-temperature single-crystal X-ray diffraction data. To obtain unbiased positions and thermal parameters for the H atoms we have also collected single-crystal neutron diffraction data. The X–N procedure to determine experimental EDs has been shown to give very accurate results (Coppens, 1967 ▸; Figgis *et al.*, 1993 ▸; Iversen *et al.*, 1997 ▸; Overgaard *et al.*, 2001 ▸). In this work, we try to rationalize the semiconducting properties of rubrene based on a topological analysis of the ED and subsequent quantitative analysis of the chemical bonds in the structure, as well as using experimentally derived energies of selected intermolecular interactions and the total lattice energy. The results are supported by extensive theoretical calculations.

## 2. Experimental   

### 2.1. Materials and crystal growth   

Fine powders of rubrene (≥ 98%) and *p*-xylene solvent (≥ 98%) were procured from Sigma–Aldrich and used without further purification. 0.2 g of rubrene was added to 20 ml of *p*-xylene under continuous stirring in an Ar atmosphere in darkness. The solution was heated to 333 K and kept at this temperature for 10 h. Later the solution was cooled to the saturation point of 318 K at the rate of 1 K h^−1^. Subsequently it was cooled to room temperature at 0.5 K h^−1^. This procedure produced good quality single crystals of various sizes. The temperature profile of crystal growth and optical micrograph of the obtained crystals are given in the supporting information (Figs. S1 and S2).

### 2.2. X-ray data collection   

Single-crystal X-ray diffraction datasets were collected on a conventional X-ray diffractometer at Aarhus University as well as at the BL02B1 beamline at the SPring8 synchrotron in Japan. For the conventional data, a high-quality single crystal with dimensions 0.24 × 0.22 × 0.18 mm was selected under a polarizing microscope and mounted using Paratone-N oil on an Agilent Technologies SuperNova diffractometer fitted with a microfocus Mo *K*α X-ray tube. The crystal was cooled to 100 K at a rate of 60 K h^−1^ using an Oxford Cryosystems Cryostream 700. High-resolution X-ray data up to (sin θ/λ)_max_ = 1.1 Å^−1^ with high redundancy (∼ 10) and completeness (∼ 100%) were obtained. The complete details of data collection and reduction procedures were published elsewhere (Jørgensen *et al.*, 2014 ▸). Synchrotron X-ray data was collected to a resolution of (sin θ/λ)_max_ = 1.51 Å^−1^ at 20 K on a single crystal of maximum dimension 0.10 × 0.09 × 0.08 mm using a wavelength of 0.35312 Å. The BL02B1 beamline is equipped with a Rigaku kappa diffractometer and a cylindrical image-plate detector. Integration of all Bragg reflections and Lorentz–polarization corrections were carried out with the software *RAPID-AUTO* (Rigaku, 2004 ▸). Sorting, scaling, merging and empirical absorption correction were carried out using the *SORTAV* program (Blessing, 1995 ▸). The crystal structure was solved by direct methods in *SHELXS* (Sheldrick, 2008 ▸) and refined using *SHELXL*97 (Sheldrick, 2008 ▸) in the *WinGX* package (Farrugia, 2012 ▸). All H atoms were located from the difference-Fourier analysis. Full crystallographic details are listed in the supporting information (Table S1).

### 2.3. Neutron data collection   

Single-crystal neutron diffraction data on rubrene were collected at 100 K using a block-shaped crystal with dimensions 1.5 × 1.5 × 1.0 mm on the single-crystal time-of-flight Laue diffractometer, TOPAZ, located at the Spallation Neutron Source at Oak Ridge National Laboratory. The integration of collected data was carried out using the program *Mantid* (Taylor *et al.*, 2012 ▸; Schultz *et al.*, 2014 ▸). The incident beam spectrum and detector efficiency corrections were performed in the program *ANVRED* (Schultz *et al.*, 1984 ▸). The crystal structure was refined using the *GSAS* program (Larson & Von Dreele, 1994 ▸; Toby, 2001 ▸). Detailed description of the data collection and reduction procedures have been reported elsewhere (Jørgensen *et al.*, 2014 ▸).

### 2.4. Computational details   

Gas-phase quantum-mechanical simulations were performed at the experimental geometry using the B3LYP (Becke, 1993 ▸) functional with 6-311G(d,p) basis set using the *GAUSSIAN*09 package (Frisch *et al.*, 2009 ▸). The topological analysis of the ED, ρ(**r**), was performed with a modified version of the program package *PROAIM* (Bieglerkonig *et al.*, 1982 ▸). Basis-set superposition error (BSSE) corrected interaction energies of molecular dimers at the crystal geometry were also evaluated. Periodic quantum-mechanical simulations at the experimental geometry were performed with the Linear Combination of Gaussian-Type Functions (LCGTF) approach as implemented in *CRYSTAL*14 (Dovesi *et al.*, 2014 ▸) at the B3LYP/6-31G(d,p) level of theory. The reciprocal space was sampled with a 4 × 4 × 4 grid in the irreducible Brillouin zone. A 30% mixing of the Fock matrices was applied to accelerate convergence, while the tolerances determining the level of accuracy of the Coulomb and exchange series were set to 10^−7^ (ITOL1 to ITOL4) and 10^−14^ (ITOL5). Theoretical structure factors with the same indices as observed in the respective experiment were computed separately and employed to derive a theoretical multipole-projected ED distribution in *XD*2006 (Volkov *et al.*, 2006 ▸). Furthermore, the topological analysis and the evaluation of the integral properties were computed directly on the LCGTF ED using the TOPOND (Gatti *et al.*, 1994 ▸) package interfaced with the CRYSTAL14 code. The experimentally obtained geometry was used as input for the calculation of the lattice energy and intermolecular interaction energy using the PIXELC module of the *CLP* computer program package (version June 2013; Gavezzotti, 2011 ▸). For this purpose, an accurate ED of the molecule was obtained independently by the MP2 and B3LYP calculations with a 6-31G(d,p) basis set in the *GAUSSIAN*09 package (Frisch *et al.*, 2009 ▸). The interaction energies of the selected molecular pairs were extracted from the analysis of crystal packing along with involved intermolecular interactions using the.mlc file generated by the PIXEL calculations. The contribution of Coulombic, polarization, dispersion and repulsion components were obtained for both the lattice energy and total intermolecular interaction energies.

### 2.5. Electron density models   

Aspherical ED features were modelled using the Hansen–Coppens multipole formalism (Hansen & Coppens, 1978 ▸) implemented in *XD*2006 (Volkov *et al.*, 2006 ▸). The core and valence scattering factors in the model were based on the wavefunctions derived by Su, Coppens and Macchi (Su & Coppens, 1998 ▸; Macchi & Coppens, 2001 ▸). The C—H bond distances were constrained to the values obtained from the structural model refined against the neutron data (*see above*). For the 100 K model the anisotropic displacement parameters (ADPs) for the H atoms were obtained from the neutron experiment. The used ADPs of H atoms were scaled based on a least-squares fit between the ADPs of the C atoms from the X-ray ED model and the neutron model, respectively, using the program *UIJXN* (Blessing, 1995 ▸). In the absence of neutron data measured at 20 K, the ADPs for hydrogen at this temperature were estimated using the *SHADE*2 webserver (Madsen, 2006 ▸), where the C—H bond distances were constrained to the values used in the 100 K ED model. In order to compare the ED results from the two temperatures, the same multipole modelling procedure was followed for both datasets and a detailed description of multipole modelling can be found elsewhere (Jørgensen *et al.*, 2014 ▸). The topological analysis of the ED was carried out in the framework of Bader’s Quantum Theory of Atoms in Molecules (QTAIM) (Bader, 1990 ▸). The NCI analysis in rubrene was carried out on the experimentally obtained ED using the NCImilano program (Saleh *et al.*, 2013 ▸). The multipole models for both datasets were examined by the Hirshfeld rigid bond test (Hirshfeld, 1976 ▸) to confirm successful deconvolution of thermal and electronic effects. The largest differences of mean-square displacement amplitudes (DMSDA) of all covalent bonds involving non-hydrogen atoms were found to be 3 × 10^−4^ Å^2^ for the C3—C4 bond in both 100 K and 20 K ED models. The minimum and maximum residual ED peaks in the multipole model [calculated for *I* > 3σ(*I*)] were −0.18 and 0.18 e Å^−3^ at 100 K and −0.19 and 0.23 e Å^−3^ at 20 K. In addition, normal probability plots, variation of scale factor with resolution, and the fractal dimension plots of the residual densities were used to confirm the high quality of the ED models (see the supporting information and our previous publication; Jørgensen *et al.*, 2014 ▸; for more details). The ADPs obtained for non-H atoms from the neutron-diffraction data and multipole model against high-resolution X-ray data at 100 K were compared to gauge the quality of the obtained datasets. The ADPs refined against the two 100 K datasets were found to be in excellent agreement with minimum deviations in mean ADPs and among the smallest mean average differences, 〈|Δ*U*|〉, ever reported for an organic compound at liquid N_2_ temperatures (Morgenroth *et al.*, 2008 ▸). To validate the experimental ED results, theoretical calculations were performed on the geometry obtained from the multipole models. The topological parameters obtained for all covalent bonds from the experiment are in good agreement with theoretical values (Table S2 in the supporting information). It is noteworthy that the agreement between theory (multipole projected) and experiment for the bond topology consistently is better for the 20 K data than for the 100 K data. Thus, for the C—C bonds the average difference of the electron density at the bond critical point is 〈Δρ_C—C_〉 = 0.053 e Å^−3^ at 20 K and 0.099 e Å^−3^ at 100 K. For the C—H bonds the values are 〈Δρ_C—H_〉 = 0.035 e Å^−3^ at 20 K and 0.060 e Å^−3^ at 100 K. This also indicates that the improved accuracy of the thermal deconvolution at 20 K is more important than the lack of unbiased neutron ADPs for the H atoms at 20 K. In general, the most accurate experimental EDs for organic crystals can be obtained at the lowest possible temperature using the X–N procedure.

## 3. Results and discussion   

### 3.1. Crystal structure   

The crystal structure and molecular packing in the crystalline state is well documented for rubrene in the literature (Jurchescu *et al.*, 2006 ▸). The asymmetric unit in the orthorhombic polymorph of rubrene is constituted by just one quarter of the whole molecule, which obeys 2/*m* symmetry (Fig. 1 ▸ *a*). A twofold axis is located along the C1—C1*b* bond and the inversion center coincides with the middle of the C1—C1*b* bond such that a mirror plane is perpendicular to the tetracene backbone of the molecule. The maximum deviation from planarity of the tetracene backbone is found to be 0.0423 (1) Å for C2 at 100 K and 0.0274 (1) Å for C4 at 20 K. The packing of the rubrene molecules generate a herringbone motif in the crystal lattice. At 100 K, the π-stacking (C_π_⋯C_π_ interaction) distance between two adjacent parallel molecules is 3.706 (1) Å and the interlayer distance perpendicular to the π-stacks is 13.875 (1) Å (see Fig. 1 ▸ *b*). There are no significant changes in these distances at 20 K [3.694 (1) and 13.868 (2) Å]. The π-stacking in orthorhombic rubrene is characterized by the absence of slippage along the *b* axis (parallel displacement), which is otherwise commonly found in the packing motifs of crystalline tetracene, pentacene and other polymorphs of rubrene (da Silva *et al.*, 2005 ▸; Bergantin & Moret, 2012 ▸; Delgado *et al.*, 2009 ▸). In a recent Hirshfeld surface (HS) analysis study of rubrene (Bergantin & Moret, 2012 ▸), these features have been clearly highlighted and described as one of the key factors for the observed semiconducting properties. Due to the herringbone packing of molecules, C5—H5⋯C4 interactions arise between tetracene backbones and C10—H10⋯C9 interactions between the phenyl rings in the crystal structure (see Fig. 1 ▸ *c*). In addition, several H⋯H interactions are observed in the crystal structure, which play a significant role in the packing of molecules (listed in Table 1 ▸). One of the significant H—H bonds is the homopolar short C8—H8⋯H8−C8 interaction with H⋯H distance of 2.2639 (1) Å. The direction of the H8—H8 bond is perpendicular to that of the C_π_⋯C_π_ stacking interactions and it is a major structure stabilizing interaction in the perpendicular direction of tetracene backbone.

### 3.2. Topological analysis   

**Figure 2:**
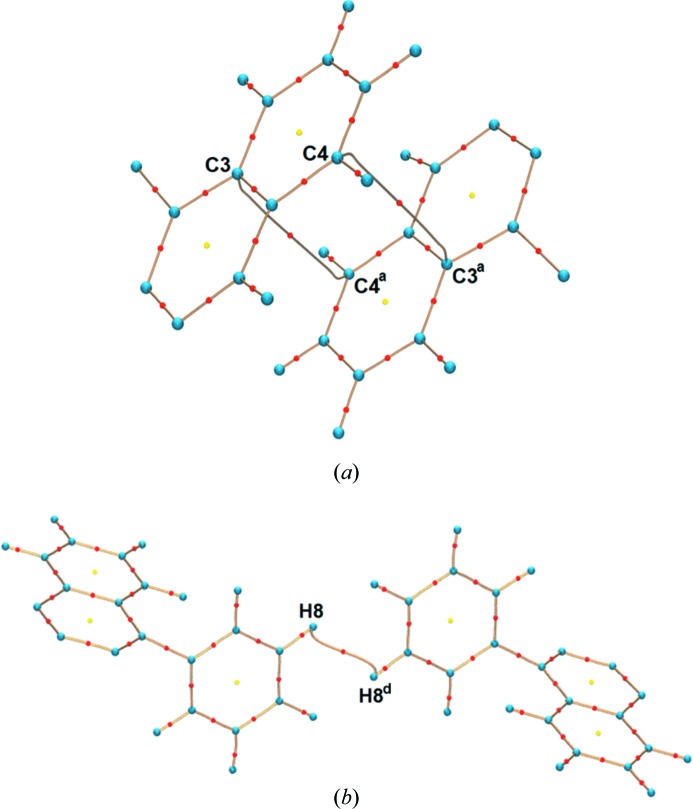
Molecular graphs from the experimental ED at 100 K showing (*a*) C_π_⋯C_π_ stacking interaction between the tetracene backbones and (*b*) H8—H8 bond. Red and yellow dots indicate b.c.p.s and ring critical points, respectively. The solid brown line separates adjacent atomic basins. Symmetry operations are listed in Table 1 ▸.

**Figure 3:**
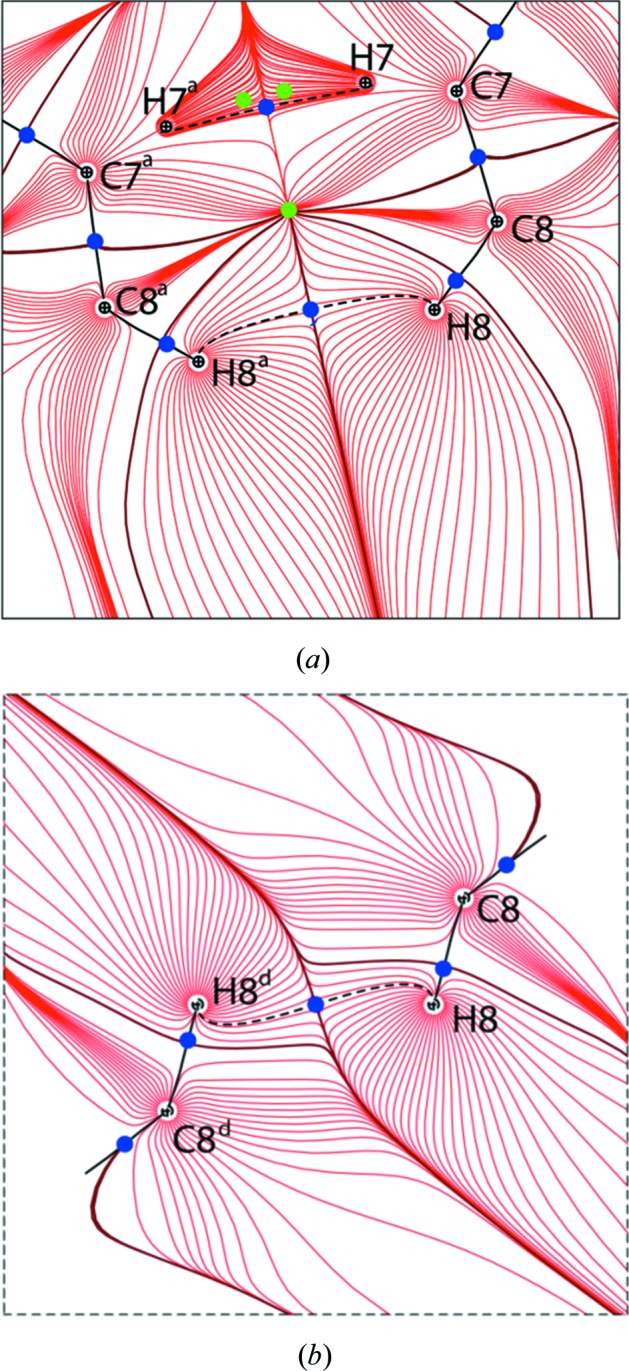
Density gradient trajectory plots demonstrating (*a*) C8—H8⋯H8—C8 and C7—H7⋯H7—C7 interactions along the tetracene backbone stacking. (*b*) C8—H8⋯H8—C8 interaction perpendicular to the tetracene backbone stacking. Heavy and dashed black lines indicate intra- and intermolecular bond paths, respectively. Adjacent atomic basins are separated by the solid brown line. The cage critical points (3, −3), b.c.p.s (3, −1) and ring critical points (3, +1) are shown as black, blue and green colored dots, respectively. Symmetry operations are listed in Table 1 ▸.

The values of the ED, Laplacian and derived properties at the bond critical points (b.c.p.s) for C_π_⋯C_π_, C—H⋯C and H⋯H interactions are listed in Table 1 ▸. The small values of ρ_bcp_ and positive ∇^2^ρ_bcp_ indicate the closed-shell nature of these interactions in the crystal structure and these values are similar to literature values (Wolstenholme *et al.*, 2007 ▸; Wolstenholme & Cameron, 2006 ▸; Zhurova *et al.*, 2006 ▸; Nguyen *et al.*, 2012 ▸; Shishkina *et al.*, 2013 ▸). The estimated potential (*V*) and kinetic (*G*) energy densities at the b.c.p.s provide additional information to classify the interactions as shared or closed shell interactions. The |*V*|/*G* ratio is smaller than 1 for closed shell interactions and larger than 2 for shared shell interactions (Espinosa *et al.*, 2002 ▸). In the case of rubrene, the |*V*|/*G* ratios are smaller than 1 for all intermolecular interactions thus, suggesting that all are closed shell interactions (Table 1 ▸). The C_π_⋯C_π_ and C⋯H interactions have comparable values of ρ_bcp_ and ∇^2^ρ_bcp_ at the b.c.p.s. The stabilizing contribution of H⋯H interactions to the energy of the crystal has been well established by the QTAIM analysis in several organic crystals (Matta *et al.*, 2003 ▸; Echeverría *et al.*, 2011 ▸; Grabowski *et al.*, 2007 ▸; Paul *et al.*, 2011 ▸). All possible H⋯H interactions which shows a b.c.p. (even when the H⋯H interaction distance is larger than the sum of the van der Waals radii equal to 2.4 Å) were considered for the topological analysis in the rubrene ED model. For H⋯H interactions, the values of ρ_bcp_ and ∇^2^ρ_bcp_ are found in the range 0.011−0.054 e Å^−3^ and 0.156–0.764 e Å^−5^, respectively, in the ED model at 100 K. For the 20 K ED model, they are in the range 0.011–0.074 e Å^−3^ and 0.162–0.785 e Å^−5^ indicating only minor differences between the two models. The obtained topological values are overall in agreement with the literature results obtained from the other experimental ED studies (Wolstenholme & Cameron, 2006 ▸; Wolstenholme *et al.*, 2007 ▸; Zhurova *et al.*, 2006 ▸; Paul *et al.*, 2011 ▸). Of all the H—H bonds, the H8—H8 bond (perpendicular to the π-stacking interactions) has the shortest bond path, 2.2688 (1) Å, and it is considerably smaller than the sum of the van der Waals radii of hydrogen. This observation is supported by the larger values of ρ_bcp_ and ∇^2^ρ_bcp_ for the H8—H8 in comparison with the rest of the H—H interactions listed in Table 1 ▸. The bond path with b.c.p.s (Fig. 2 ▸) and density gradient trajectory plots (Fig. 3 ▸) further illustrate these interactions in the crystal packing. It has already been shown that H—H bonding supplement the van der Waals interactions in the crystal by the detailed evaluation of the ED, Laplacian and energy densities at CPs as a function of the bond distance (Paul *et al.*, 2011 ▸). They demonstrated that H—H bonding follows the exponential relations between the kinetic and potential energy densities (*G* and *V*) with the bond distance and the linear dependence of the total energy density (*H*) on the positive Hessian curvature, which is similar to the nature of the van der Waals interactions. In this study, the intermolecular bond paths for the C_π_⋯C_π_ and H—H bonding are longer than the direct inter-nuclear distances as they are curved (see Fig. 3 ▸). Curved bond paths are common in interactions involving π-electron density (Lu *et al.*, 2007 ▸; Macchi *et al.*, 1998 ▸; Scherer *et al.*, 2006 ▸) and weak closed shell interactions like H—H bonding (Wolstenholme *et al.*, 2007 ▸). Integrated net atomic charges are calculated from the QTAIM analysis and they are listed in Table S3. A small positive (δ^+^) charge on all hydrogen atoms indicates that all H—H bonds correspond to homopolar H^δ+^⋯H^δ+^ interactions. Additionally, both the 100 K and the 20 K ED models yield similar values for the derived topological properties at the b.c.p. for all covalent bonds and intermolecular interactions based on the QTAIM analysis. The topological analysis of the ED could establish the presence of C_π_⋯C_π_ stacking interactions between the adjacent rubrene molecules in the crystalline state, but there is no direct relationship available to connect the topological features with the semiconducting properties like mobility in rubrene.

### 3.3. Lattice energy and interaction energies   

Calculation of lattice energy and interaction energies of molecular dimers using the PIXEL (Gavezzotti, 2011 ▸) method enables partitioning of the total energy into electrostatic, polarization, dispersion and repulsion components. The values obtained from the PIXEL calculations are known to be comparable to high level MP2 and DFT-D quantum mechanical calculations (Gavezzotti, 2008 ▸; Dunitz & Gavezzotti, 2012 ▸; Maschio *et al.*, 2011 ▸; Braun *et al.*, 2013 ▸). The different energy contributions reveal that a significant amount of the total lattice energy comes from the dispersion interactions (*E*_disp_ ≃ 5.6*E*_es_ ≃ 9.1*E*_pol_, Table 2 ▸). To evaluate the importance of non-covalent interactions in the crystal packing, interaction energies between selected molecular dimers were estimated using the experimental ED model geometry (Table 2 ▸). The primary interacting dimers in the crystal structure are formed through the following interactions: C*_π_*⋯C*_π_* stacking (dimer I), C—H⋯C (dimer II), and H—H interactions (dimers III and IV). Not surprisingly, the dispersion interaction energy is the largest contributor to the total interaction energy of all molecular dimers. The interaction energy of the molecular pair connected by C*_π_*⋯C*_π_* interaction (dimer I) along the crystallographic *b* axis is much larger compared with the interaction energy of the molecular pair connected by the H—H bonding (dimer III) along the crystallographic *a* axis. The interaction energy of the molecular dimer with C—H⋯C interaction (dimer II) in the herringbone packing motif constitute a significant amount of interaction energy (∼ −48.5 kJ mol^−1^) in the crystal packing (Tables 2 ▸ and 3 ▸). It is important to notice that these C—H⋯C interactions do not cause any slippage of the π⋯π stacking along the *b* axis. Besides these interactions, a significant contribution to the interaction energy (∼ −6.6 kJ mol^−1^) is provided by molecular pairs with the H9⋯H9 bonding (dimer IV) in the crystal packing. The rest of the H—H interactions listed in Table 1 ▸ do not contribute significantly to the interaction energies in the crystal structure (∼ 0–0.5 kJ mol^−1^). There are no significant changes found in the PIXEL interaction energies using the crystal geometry obtained by the ED models at 100 K and 20 K. To estimate the influence of computational methods on the interaction energy, the ED for the PIXEL calculations is also obtained from the MP2/6-31G(d,p) calculations and obtained energies are listed in Table S4. We have found that there are no significant deviations in the estimated energies using the ED obtained from the DFT and MP2 calculations in PIXEL.

In addition to the PIXEL calculations, the lattice energy and intermolecular interaction energies for selected molecular pairs were calculated from the ED model using the XDPROP module in *XD*2006 (Volkov *et al.*, 2006 ▸). The resulting total energy is composed of electrostatic, exchange–repulsion and dispersion terms. The electrostatic term is estimated using the Exact Potential and Multipole Method (EP/MM) (Volkov *et al.*, 2004 ▸), while the exchange–repulsion and dispersion terms are approximated by Williams and Cox atom–atom potentials (Williams & Cox, 1984 ▸). Obtained values from the ED models and corresponding theoretical models are listed in Table 3 ▸. The derived energy values from the ED models are in good agreement with the PIXEL values for all interacting molecular dimers (I to IV), whereas the lattice energy deviates significantly in the ED models (see Tables 2 ▸ and 3 ▸). In comparison with the PIXEL values, the maximum deviation of ∼ 7 kJ mol^−1^ was observed for the molecular dimer with C*_π_*⋯C*_π_* interactions (dimer I) in the theoretical ED model at 100 K. In contrast, a difference of ∼ 44 kJ mol^−1^ was found for lattice energies obtained in the ED models. The two methods use different schemes to evaluate lattice and intermolecular dimer energies and significant differences arise in the estimation of polarization energy contribution to the total energy (Gavezzotti, 2011 ▸; Volkov *et al.*, 2004 ▸). In the multipole model (both experimental and theoretical models), the ED of one rubrene molecule is computed within the crystal, and therefore it inherently contains effects of polarization from the surrounding molecules in the crystal. Thus, the electrostatic energy (*E*_es_) calculated for a pair of molecules is the sum of the unperturbed electrostatic interaction between the two molecules along with the polarization contributions due to the entire crystal. The *E*_es_ and *E*_pol_ quantities cannot be retrieved separately from each other in the multipole method. However, in the PIXEL calculation, the electrostatic energy is that of the unperturbed molecules and the polarization energy is the mutual interaction of just the two molecules of each dimer. The *E*_pol_ energy does not include the polarization contributions from the surrounding molecules in the crystal. Hence, the polarization energy contributes significantly to the observed deviations in the energy values from the PIXEL and multipole methods. Further, the small differences in the *E*_es_ contribution from the experimental and theoretical ED models is due to the multipole populations of atoms in the multipole model. The electrostatic energy calculation in the EP/MM method depends on the multipole parameters of the ED model (Volkov *et al.*, 2004 ▸). The slightly different multipole populations result in a deviation of estimated electrostatic energy in the ED model. Similarly, a detailed analysis of the discrepancy in estimating lattice energies from different multipole refinement models and thermal motion analysis for H atoms was recently reported in a study of sulfathiazole polymorphs (Sovago *et al.*, 2014 ▸). In this study, there are no significant deviations in the calculated energies from both the experimental and theoretical ED models at 100 K and 20 K using the EP/MM method. It is important to note that the entropic effects on energetics of intermolecular interactions are not accounted for in the comparison of energetics calculated using the 100 K and 20 K ED models. Further, the use of different schemes for the estimation of dispersion energy in PIXEL and XD methods also contributes to differences in the obtained lattice and interaction energies. To study the effect of the hydrogen modeling on the interaction energy of dimers, we refined a 100 K model using the ADPs of hydrogen obtained from the SHADE2 webserver (Madsen, 2006 ▸), *i.e.* an identical approach as the 20 K ED model. However, this did not change the interaction energy values of dimers significantly.

### 3.4. Non-covalent interactions (NCI) analysis   

**Figure 4:**
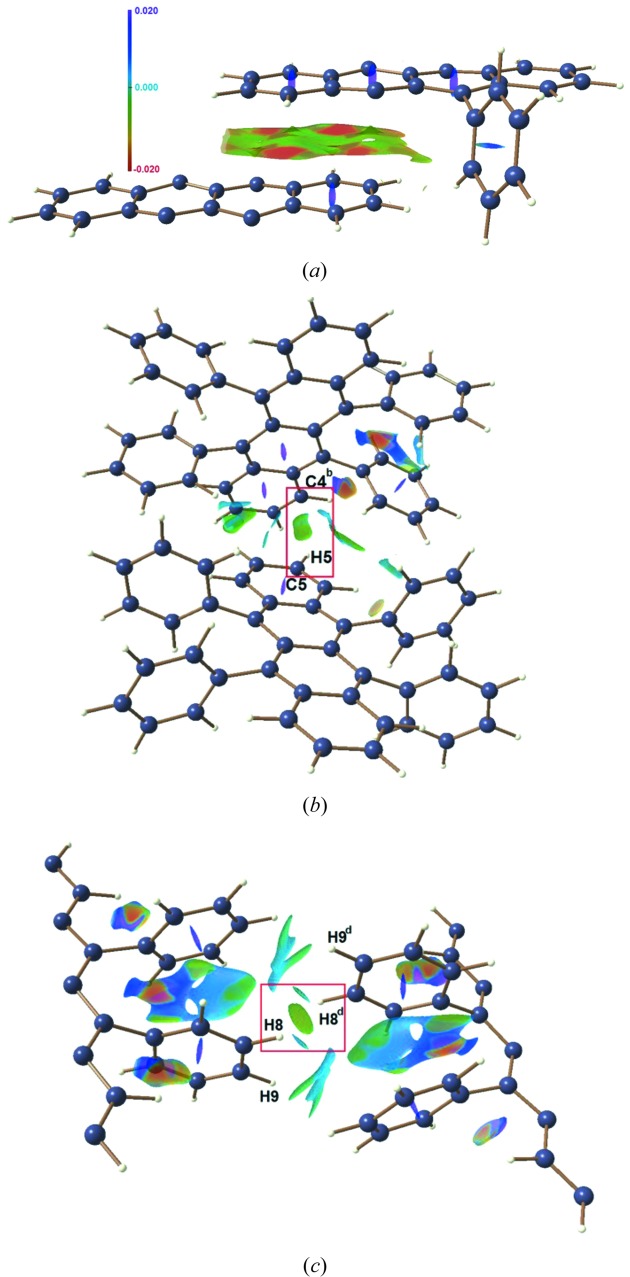
RDG-based NCI isosurfaces obtained from the experimental ED model at 100 K for (*a*) C_π_⋯C_π_ stacking interactions, (*b*) C4⋯H5 interaction and (*c*) homopolar H8—H8 bond. NCI isosurfaces corresponding to RDG = 0.6 a.u. The surfaces are colored on a red–green–blue–purple scale [−0.020 < sign(λ_2_)ρ < 0.020 a.u.]. Red, green and blue/purple indicate stabilizing, intermediate and destabilizing overlap regions, respectively. Symmetry operations are listed in Table 1 ▸.

The electron density, ρ(*r*), between interacting atoms can be determined by the experimental measurements and theoretical calculations. The reduced density gradient [RDG = 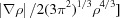, a dimensionless quantity, is derived using the ED and its first derivative in real space (Johnson *et al.*, 2010 ▸; Saleh *et al.*, 2012 ▸). Low ED and low RDG values normally correspond to the non-covalent interactions (NCIs) between two interacting atoms. The NCI descriptor based on sign(λ_2_)ρ(*r*) is useful to characterize NCIs at each RDG isosurface point, where λ_2_ is the second largest eigenvalue of the ED Hessian matrix. The mapping of the quantity, sign(λ_2_)ρ(*r*) on RDG isosurfaces can distinguish stabilizing [sign(λ_2_)ρ(*r*) < 0] and destabilizing [sign(λ_2_)ρ(*r*) > 0] interactions. Here, they have been visualized by plotting the RDG isosurfaces using the *MolIso* program (Hubschle & Luger, 2006 ▸) see in Fig. 4 ▸. Red isosurfaces correspond to stabilizing interactions and blue isosurfaces represent steric repulsive interaction regions. The C*_π_*⋯C*_π_* stacking interactions [*d* = 3.708 (1) Å at 100 K] between adjacent rubrene molecules is clearly seen as the isosurfaces filling the interlayer spaces between tetracene backbones in Fig. 4 ▸(*a*) obtained from the experimental ED model at 100 K. The observation of low density and low RDG isosurfaces between the stacked aromatic rings indicate the overall balance of steric (destabilizing) and dispersive (stabilizing) contributions (Johnson *et al.*, 2010 ▸; Saleh *et al.*, 2012 ▸). The red and green regions on the NCI isosurfaces in Fig. 4 ▸(*a*) correspond to negative sign(λ_2_)ρ(*r*) values, which indicate the presence of stabilizing contributions provided by C_π_⋯C_π_ interactions between the tetracene backbones. This is also supported by the QTAIM analysis where topological properties correspond to closed shell van der Waals interactions with significant contributions to the total interaction energy of molecular pairs connected by C_π_⋯C_π_ interactions coming from the dispersion energy (Table 1 ▸, dimer I in Tables 2 ▸ and 3 ▸). Additionally, the large surface area of the NCI isosurface corresponds to the delocalized nature of C_π_⋯C_π_ stacking interactions. The NCI surfaces were related to the b.c.p.s of interaction obtained by the QTAIM analysis in the literature (Lane *et al.*, 2013 ▸; Saleh *et al.*, 2012 ▸) to obtain a global description of chemical bonding by the NCI analysis. In Fig. 4 ▸(*a*), the red and green isosurface corresponds to the regions surrounding the b.c.p. of C*_π_*⋯C*_π_* stacking interaction, whereas the blue isosurface coincides with ring critical points of the aromatic ring. The nature of π⋯π interactions has been explored by a large number of experimental and theoretical studies (Hunter & Sanders, 1990 ▸; Hunter *et al.*, 2001 ▸; Meyer *et al.*, 2003 ▸; Sinnokrot *et al.*, 2002 ▸). Hunter & Sanders (1990 ▸) proposed a model to describe the nature of π⋯π interactions in porphyrin where the π⋯π interactions was favourable due to π–σ interactions that overcome π–π repulsions. However, when a large surface area is available for stacking interactions, van der Waals interactions and desolvation contributions are also very important in the stability of the π⋯π interactions (Meyer *et al.*, 2003 ▸). The red and green regions in the isosurfaces of the π⋯π interaction (Fig. 4 ▸ *a*) indicate that the contribution of stabilizing interactions surpasses those of destabilizing interactions in the π–π stacking. This stacking interaction is expected to improve charge transport properties along the stacked aromatic layers as confirmed by good OFET characteristics observed in the direction of stacking layers. The pivotal role of aromatic π⋯π interactions in the formation of a molecular bridge between adjacent molecules has been experimentally demonstrated by molecular conductance measurements in oligo-phenylene ethynylene–monothiol molecules (Wu *et al.*, 2008 ▸), conjugated polymer poly(3-hexylthiophene) (Sirringhaus *et al.*, 1999 ▸) and phenylene vinylene derivatives (Seferos *et al.*, 2005 ▸). The strong π–π coupling through intermolecular π⋯π interactions between molecular junctions is responsible for the efficient charge transport across the molecular junction and it was successfully demonstrated by the measurement of charge transport properties across the molecular junction (Wu *et al.*, 2008 ▸; Sirringhaus *et al.*, 1999 ▸; Seferos *et al.*, 2005 ▸). Furthermore, the confirmation of π⋯π interactions from the NCI analysis correlate well with the topological properties obtained by the QTAIM analysis. Additionally, it supports earlier theoretical predictions (Wen *et al.*, 2009 ▸; Kobayashi *et al.*, 2013 ▸; Stehr *et al.*, 2011 ▸) and single-crystal FET characteristics (Podzorov *et al.*, 2004 ▸), where an anisotropic charge transfer was observed for rubrene with the highest charge mobility along the crystallographic *b* axis due to the stacking interactions between the tetracene backbones. RDG isosurfaces for the C4⋯H5 interaction are highlighted in Fig. 4 ▸(*b*). The green RDG isosurface is localized between the C and H atoms demonstrate the presence of C⋯H interactions in the crystal packing. A green oblate RDG isosurface in Fig. 4 ▸(*c*) is found for the homopolar C8—H8⋯H8—C8 interaction (perpendicular to the C_π_⋯C_π_ stacking interactions). The two smaller isosurface discs seen close to the larger RDG isosurface of the H8—H8 bonding correspond to subtle C9—H9⋯H8—C8 interactions [2.745 (2) Å] in Fig. 4 ▸(*c*). This distance is much longer than the sum of the van der Waals radii of H—H bonding (2.4 Å) and has much smaller contribution to the crystal packing. These observations are also supported by interaction energy calculations using the PIXEL and multipole methods (*see above*). The NCI analysis complements the QTAIM analysis. In the latter approach, only a limited number of bcps corresponding to C⋯C interactions was found, while in the NCI, the low values of the RDG indicate significant intermolecular interactions (Fig. 4 ▸ *a*). 

Even though the electron density study does not provide direct information on the observed physical properties of rubrene, it is still useful to experimentally establish the factors such as intermolecular interactions, which are responsible for the semiconducting properties in the solid state. In the solid state, the packing of molecules dictates interesting physical and chemical properties of the compound. Topological properties, NCI analysis and energetics of intermolecular interactions further confirm the nature and strength of chemical interactions in governing the close packing of molecules in the crystalline lattice. It is interesting to note that there is very little qualitative or quantitative change in the intermolecular interactions in rubrene when the temperature is changed from 20 K to 100 K. Podzorov *et al.* (2004 ▸) reported the carrier mobility in rubrene crystals as a function of temperature and observed two distinct regimes below and above 150 K. In the temperature range 150–300 K the mobility is intrinsic and highly anisotropic with the mobility along the *b*-axis being 2–3 times larger than along the *a*-axis. This supports the significance of the π⋯π interactions for the electron transport. From 100 K to 150 K the transport mechanism changes, and the mobility becomes almost isotropic and dominated by defect traps. In the present study it is shown that the electron density at 20 K and 100 K is virtually unchanged, so if transport data could be measured below 100 K, then any potential change in mechanism is unlikely to be be due to changes in the intrinsic properties of rubrene.

## 4. Conclusions   

Using high-resolution X-ray and neutron diffraction data, a high-quality X–N ED model was obtained for rubrene at 100 K. Furthermore, high-resolution synchrotron X-ray data up to (sinθ/λ)_max_ = 1.51 Å^−1^ were collected at 20 K to obtain precise ED distributions with minimum perturbations from thermal vibration of atoms. Comparison of the results obtained at 20 K and 100 K, respectively, confirms that the most accurate experimental EDs for organic molecules can be obtained at the lowest possoible temperature using the X–N procedure. The combined topological properties of the ED, the NCI analysis and the interaction energies from both experimental and theoretical models confirm the presence of π⋯π stacking interactions (dimer energy of ∼ −68.5 kJ mol^−1^) between the tetracene backbones of rubrene in the crystal packing. The π⋯π stacking interactions along the *b*-axis are unaltered (no slippage) by C—H⋯C interactions (dimer energy of ∼−49.0 kJ mol^−1^) observed in the herringbone packing motif. Additionally, homopolar H—H bonds (H8—H8) between the phenyl rings display the shortest interaction distance of 2.269 (1) Å among all H—H interactions found in the crystal structure. The molecular pairs connected by H—H bonding in rubrene are found to have interaction energies in the range 0 to −24 kJ mol^−1^. The quantitative analysis of H—H interactions provides more insight into the nature of these chemical interactions in terms of derived topological properties and interaction energies. There were no significant changes in the topological properties of ED and interaction energies from the 100 K and 20 K ED models. Interaction energies of molecular dimers obtained from the ED models are in good agreement with the PIXEL values. The calculation of different energy contributions to lattice and intermolecular interaction energies demonstrate that the crystal structure and physical properties of rubrene in the solid state are mainly governed by the dispersion energy component of intermolecular interactions. The quantitative analysis of non-covalent interactions brings out the significant role of weak intermolecular interactions in dictating the crystal structure and physical properties of orthorhombic rubrene in the crystalline state.

## Supplementary Material

Crystal structure: contains datablock(s) rubrene_20K, rubrene_100K. DOI: 10.1107/S2052252515012130/lc5064sup1.cif

Structure factors: contains datablock(s) rubrene_20K. DOI: 10.1107/S2052252515012130/lc5064rubrene_20Ksup2.hkl

Structure factors: contains datablock(s) rubrene_100K. DOI: 10.1107/S2052252515012130/lc5064rubrene_100Ksup3.hkl

Supporting figures and tables. DOI: 10.1107/S2052252515012130/lc5064sup4.pdf

CCDC references: 1418604, 1025039

## Figures and Tables

**Table 1:** Topological parameters of intermolecular interactions in rubrene The values reported in the first, second and third lines correspond to the experimental multipole model, theoretical multipole model and the theoretical LCGTF from TOPOND, respectively; *G*, *V* and *H* are kinetic, potential and total energy densities at b.c.p., respectively. Errors on the experimental ^2^_b_ are not available, but combined random (least squares) and systematic (model) errors are at least on the second digit after the decimal point.

Temperature	Interaction	*R_ij_* ()	_b_ (e^3^)	^2^_b_ (e^5^)	*G* (kJmol^1^bohr^3^)	*V* (kJmol^1^bohr^3^)	*H* (kJmol^1^bohr^3^)	|*V*|/*G*
100K	C3C4 ^i^	3.7304	0.031(1)	0.22	5.0	4.0	1.0	0.81
			0.022	0.22	4.6	3.1	1.4	0.67
			0.024	0.24	5.2	4.0	1.2	0.77
	C4H5^ii^	2.8252	0.037(2)	0.27	6.3	5.2	1.1	0.83
			0.033	0.31	6.8	5.1	1.7	0.75
			0.040	0.44	9.7	7.2	2.4	0.74
	C9H10^iii^	2.8673	0.032(3)	0.21	4.9	4.0	0.9	0.83
			0.036	0.25	5.9	4.9	1.0	0.83
			0.038	0.38	8.2	6.2	2.0	0.76
	H8H8^iv^	2.2688	0.046(2)	0.57	12.3	9.1	3.2	0.74
			0.032	0.69	13.5	8.4	5.2	0.62
			0.054	0.76	15.5	10.3	5.3	0.66
	H8H8^i^	2.4026	0.024(2)	0.31	6.3	4.1	2.2	0.66
			0.023	0.28	5.8	3.8	1.9	0.66
			0.034	0.43	8.8	5.9	2.9	0.67
	H7H7^i^	2.5174	0.028(4)	0.49	9.7	6.1	3.5	0.63
			0.031	0.47	9.6	6.3	3.3	0.66
			0.035	0.46	9.1	5.8	3.3	0.64
	H4H7^i^	2.4068	0.034(3)	0.23	5.4	4.5	0.9	0.83
			0.021	0.31	6.2	3.9	2.3	0.63
			0.033	0.41	8.3	5.5	2.8	0.66
	H9H9^v^	2.6703	0.020(1)	0.21	4.4	2.9	1.5	0.67
			0.018	0.22	4.3	2.8	1.5	0.65
			0.022	0.28	5.6	3.6	2.0	0.64
	H5H11^vi^	2.1631	0.048(4)	0.33	8.1	7.2	0.9	0.89
			0.042	0.34	7.8	6.4	1.3	0.82
			0.048	0.59	12.8	9.6	3.3	0.75
	H4H11^vii^	2.6045	0.034(3)	0.39	8.2	5.9	2.3	0.72
			0.035	0.44	9.3	6.5	2.8	0.70
			0.041	0.46	9.9	7.2	2.7	0.73
	H8H9^viii^	2.7562	0.015(1)	0.17	3.4	2.1	1.3	0.64
			0.011	0.16	3.1	1.8	1.3	0.58
			0.017	0.19	3.9	2.6	1.3	0.67
20K	C3C4 ^i^	3.7334	0.025(1)	0.23	5.4	3.8	1.6	0.70
			0.023	0.22	4.7	3.3	1.4	0.70
			0.025	0.24	5.4	4.1	1.3	0.76
	C4H5^ii^	2.8192	0.039(2)	0.33	7.4	6.0	1.4	0.81
			0.033	0.32	6.9	5.1	1.7	0.74
			0.042	0.47	10.1	7.4	2.7	0.73
	C9H10^iii^	2.8577	0.041(2)	0.27	6.5	5.7	0.8	0.88
			0.036	0.26	6.1	5.0	1.1	0.82
			0.038	0.38	8.4	6.4	2.0	0.76
	H8H8^iv^	2.2639	0.074(2)	0.79	18.6	15.8	2.8	0.85
			0.035	0.66	13.3	8.5	4.8	0.64
			0.055	0.78	15.9	10.5	5.4	0.66
	H8H8^i^	2.3743	0.034(2)	0.43	9.1	6.3	2.7	0.69
			0.025	0.31	6.3	4.2	2.1	0.67
			0.035	0.45	9.2	6.2	3.0	0.67
	H7H7^i^	2.4816	0.040(3)	0.56	11.7	8.2	3.5	0.70
			0.021	0.38	7.4	4.5	2.9	0.61
			0.037	0.49	9.8	6.2	3.5	0.63
	H4H7^i^	2.4082	0.043(2)	0.37	8.6	6.9	1.6	0.80
			0.030	0.32	6.8	4.8	2.0	0.71
			0.034	0.42	8.6	5.8	2.9	0.67
	H9H9^v^	2.6288	0.021(1)	0.28	5.5	3.6	2.0	0.65
			0.021	0.45	8.6	5.1	1.7	0.59
			0.024	0.30	6.1	3.9	2.2	0.64
	H5H11^vi^	2.1565	0.040(5)	0.49	10.4	7.5	2.9	0.72
			0.043	0.35	8.1	6.7	1.4	0.83
			0.048	0.60	13.1	9.8	3.3	0.75
	H4H11^vii^	2.6102	0.044(1)	0.52	11.3	8.4	2.9	0.74
			0.041	0.46	10.0	7.4	2.6	0.74
			0.041	0.47	10.1	7.4	2.8	0.73
	H8H9^viii^	2.7996	0.011(1)	0.16	3.1	1.8	1.3	0.58
			0.014	0.17	3.4	2.1	1.3	0.62
			0.017	0.19	4.0	2.7	1.3	0.68

Symmetry codes: (i) 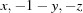; (ii) 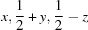; (iii) 
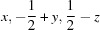
; (iv) 
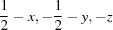
; (v) 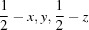; (vi) 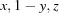; (vii) 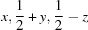; (viii) 
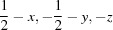
.

**Table 2:** Lattice energy and intermolecular interaction energies of selected molecular dimers (kJmol^1^) in rubrene obtained from the PIXEL calculations using the ED from the DFT method The values reported in the first and second lines correspond to the crystal geometry at 100K and 20K, respectively. Symmetry operations are listed in Table 1 ▸.

Molecular dimers	Interaction distance ()	Centroidcentroid distance ()	*E*_es_	*E*_pol_	*E*_disp_	*E*_rep_	*E*_tot_
Lattice energy			54.3	33.6	304.5	166.6	225.8
			56.6	35.0	309.8	175.1	226.3
CC stacking^i^ (dimer I)	3.706(1)	7.160(2)	8.5	13.8	110.2	62.7	69.8
	3.694(1)	7.160(2)	9.3	14.4	112.3	66.5	69.5
C4H5^ii^ (dimer II)	2.825(1)	7.953(3)	15.2	6.5	61.9	35.2	48.5
	2.817(1)	7.930(2)	15.5	6.7	62.6	36.3	48.5
H8H8^iv^ (dimer III)	2.268(1)	13.875(4)	6.6	2.7	24.2	14.6	18.8
	2.264(1)	13.868(3)	7.0	2.8	24.6	15.2	19.1
H9H9^v^ (dimer IV)	2.667(1)	15.170(2)	1.1	0.4	7.6	2.4	6.6
	2.623(1)	15.152(2)	1.2	0.5	8.1	2.9	6.9

**Table 3:** Lattice energy and intermolecular interaction energies of selected molecular dimers (kJmol^1^) in rubrene obtained using the ED models in *XD*2006 ’Theory-multipole’ values are from the multipole projection of theoretical static structure factors. Symmetry operations are listed in Table 1 ▸.

	ED model	Method	*E*_rep_	*E*_es_ (includes *E*_pol_)	*E*_disp_	*E*_tot_
Lattice energy	100K	Experimental	178.1	64.2	377.9	264.0
		Theory-multipole		67.5		267.3
	20K	Experimental	186.4	69.6	384.7	267.9
		Theory-multipole		63.4		261.7
CC stacking^i^ (dimer I)	100K	Experimental	65.7	4.1	131.7	70.1
		Theory-multipole		9.0		75.0
	20K	Experimental	69.2	4.2	134.0	69.0
		Theory-multipole		7.4		72.2
C4H5^ii^ (dimer II)	100K	Experimental	36.8	9.3	76.7	49.2
		Theory-multipole		13.9		53.8
	20K	Experimental	38.0	10.7	77.8	50.5
		Theory-multipole		13.8		53.6
H8H8^iv^ (dimer III)	100 K	Experimental	16.1	10.4	29.2	23.5
		Theory-multipole		4.1		17.2
	20K	Experimental	16.9	7.2	29.8	20.1
		Theory-multipole		4.5		17.4
H9H9^v^ (dimer IV)	100K	Experimental	3.3	0.1	8.9	5.8
		Theory-multipole		0.0		5.6
	20K	Experimental	3.8	0.1	9.4	5.5
		Theory-multipole		0.0		5.6
