# Supplementary material for: Quantitative analysis of intermolecular interactions in orthorhombic rubrene
Source: IUCrJ. 2015 Aug 14;2(Pt 5):563–74. doi: 10.1107/S2052252515012130 (PMC4547824; doi:10.1107/S2052252515012130)
Supplement: Supplementary file 4 [file m-02-00563-sup4.pdf]

IUCrJ

Volume 2 (2015)

Supporting information for article:

Quantitative analysis of intermolecular interactions in orthorhombic rubrene

Venkatesha R. Hathwar, Mattia Sist, Mads R. V. Jørgensen, Aref H. Mamakhel, Xiaoping Wang, Christina M. Hoffmann, Kunihisa Sugimoto, Jacob Overgaard and Bo Brummerstedt Iversen

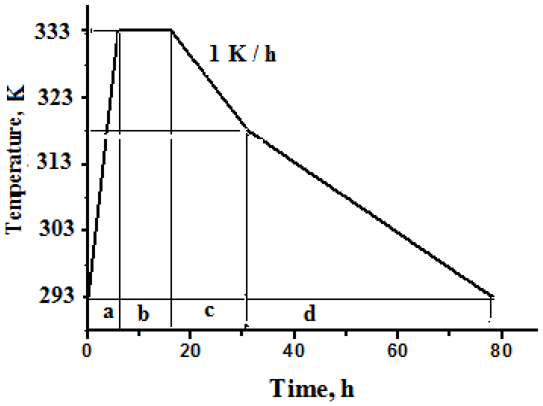

Figure S1 The temperature profile for the crystal growth of rubrene: (a) the rubrene solution temperature was raised to 333K (b) the temperature was maintained at 333K for 10h (c) the solution was cooled to the saturation point (318K) at a rate of 1K/h (d) the solution is cooled to room temperature at a rate of ≤ 0.5K/h during which the crystals form.

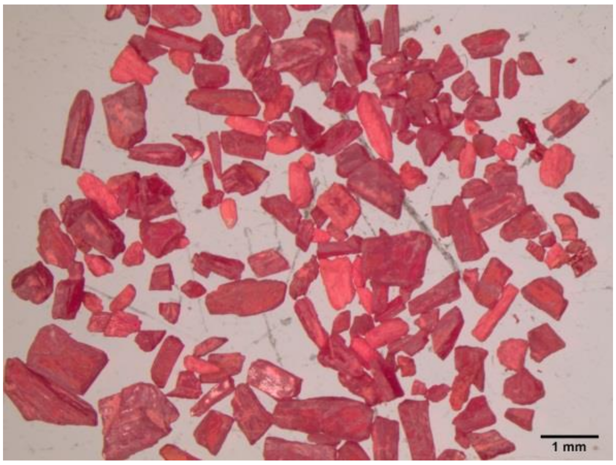

Figure S2 Optical micrograph showing the obtained single crystals of rubrene.

Table S1 Crystallographic and refinement details of 20 K synchrotron data collected at the BL02B1 beamline at SPring-8, Japan.

Radiation Source	**Synchrotron**
Empirical Formula	$C_{42}H_{28}$
Formula Weight, g mol-1	532.64
Crystal size (μm)	100
Crystal system	Orthorhombic
Space Group	Cmca
λ, Å	0.35312
a, Å	26.7965(5)
b, Å	7.1599(1)
c, Å	14.1519(3)
V, Å3	2715.19(9)
Z	4
F(000)	1120
T, K	20
ρ, g cm^{-3}	1.299
μ, mm^{-1}	0.007
T_{max}, T_{min}	1.0000, 0.9993
$\sin(\theta)/\lambda_{max}$, $Å^{-1}$	1.51
N_{meas}, N_{uniq}	441669, 19909
Redundancy	22.2
Completeness	1.000
R_{int}	0.0514
N_{obs}, N_{var}, (3σ)	14111, 384
$R(F^2)$, $wR(F^2)$ ($I>2\sigma(I)$)	0.0192, 0.0392
Goodness of fit	1.043
$\Delta\rho_{min/max}$ e$Å^{-3}$ all data, $\sin\theta/\lambda<1.1Å^{-1}$	–0.188/0.226, –0.089/0.133

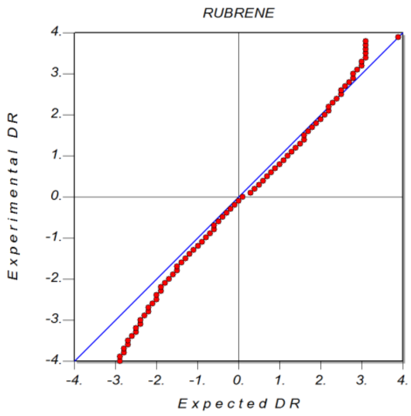

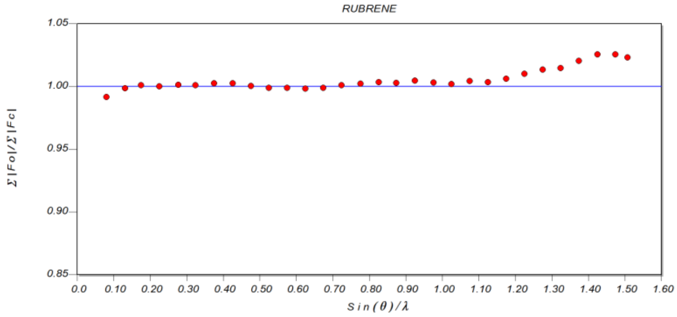

(a) (b)

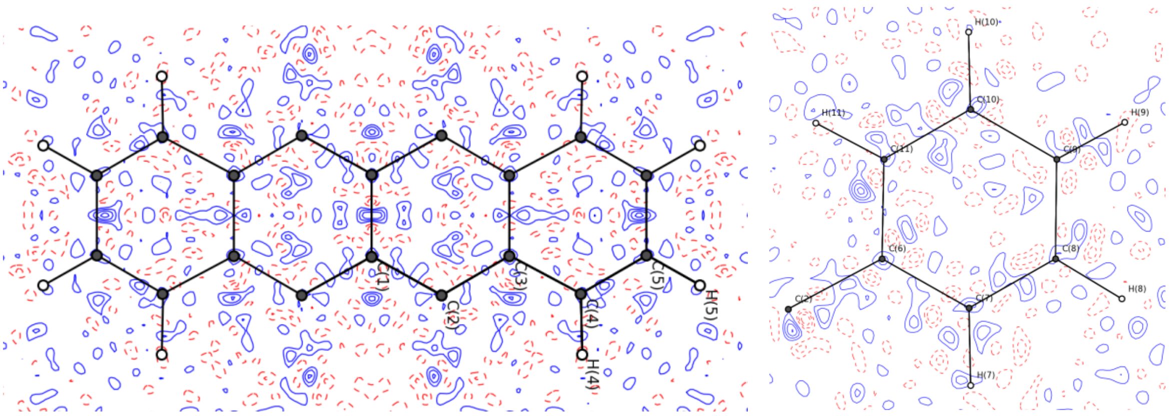

(c)

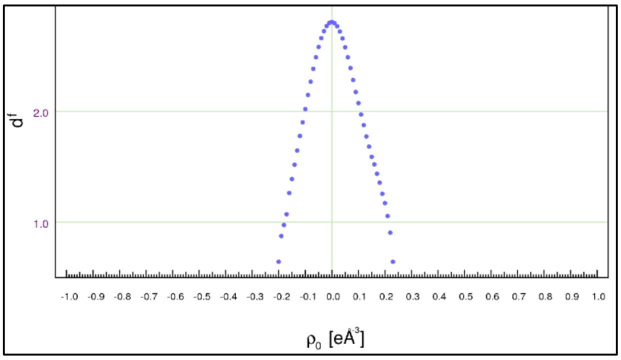

(d)

Figure S3 The quality of the ED model at 20 K was estimated by (a) the normal probability plot (b) a variation of scale factor with resolution (c) residual density maps are at ±0.05 contour intervals and (d) the fractal dimension plot. These all suggest that an excellent data quality and a good fit of the multipole model of electron density.

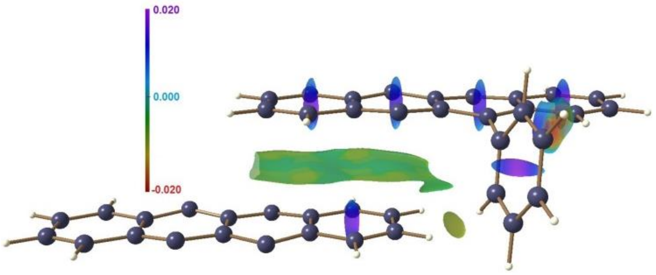

(a)

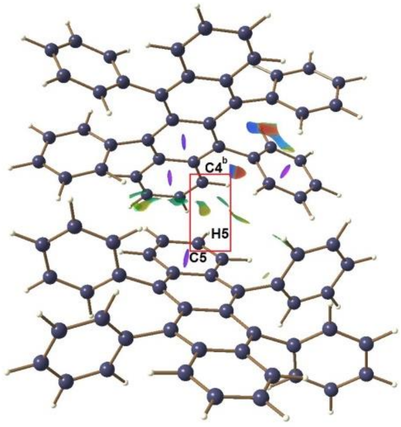

(b)

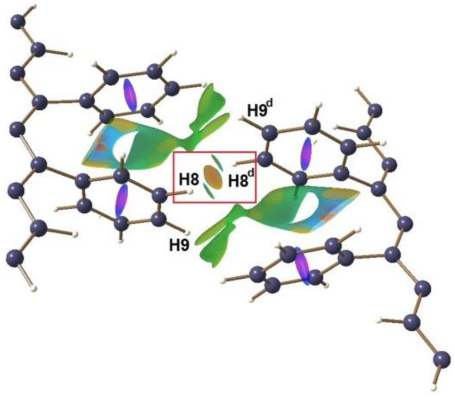

(c)

Figure S4 RDG-based NCI isosurfaces obtained from the experimental ED model at 20 K for (a) $C_\pi \cdots C_\pi$ stacking interactions (b) C4···H5 interaction and (c) homopolar H8–H8 bonds. NCI surfaces correspond to RDG = 0.6 au. The surfaces are colored on a red-green-blue scale ($-0.020 < \mathrm{sign}\,(\lambda_2)\rho < 0.034$ au). Red, green and blue indicate strong stabilizing, intermediate and destabilizing overlap regions, respectively.

Table S2 List of intra-molecular BCP's obtained from the experimental and theoretical ED models at 100 K and 20 K. The values reported in first, second and third lines correspond to the experimental multipole model, theoretical multipole model and theory from PROAIM, respectively.

Temperature	Bond	R_{ij} (Å)	ρ_b (eÅ^{-3})	$\nabla^2\rho_b$ (eÅ^{-5})	ε
100 K	C1-C2	1.4264	2.022	-18.573	0.26
			1.960	-15.190	0.20
			1.960	-18.200	0.20
	C2-C3	1.4065	2.133	-21.053	0.18
			2.035	-16.543	0.19
			2.040	-19.600	0.23
	C3-C4	1.4373	2.011	-19.129	0.14
			1.922	-15.145	0.14
			1.931	-18.200	0.14
	C4-C5	1.3658	2.290	-25.029	0.17
			2.191	-19.333	0.23
			2.192	-22.600	0.26
	C4-H4	1.0839	1.906	-24.763	0.06
			1.918	-20.889	0.03
			1.918	-23.600	0.02
	C5-H5	1.0876	1.899	-22.485	0.06
			1.889	-20.810	0.03
			1.886	-23.000	0.02
	C2-C6	1.4972	1.870	-17.167	0.01
			1.737	-11.918	0.03
			1.737	-15.000	0.03
	C6-C7	1.3979	2.156	-21.207	0.13
			2.084	-17.367	0.18

			2.065	-20.200	0.21
	C7-C8	1.3920	2.239	-24.182	0.17
			2.112	-17.966	0.20
			2.092	-20.900	0.21
	C8-C9	1.3942	2.160	-23.294	0.26
			2.096	-17.473	0.19
			2.086	-20.900	0.20
	C9-C10	1.3956	2.201	-24.165	0.14
			2.084	-17.226	0.18
			2.073	-20.500	0.20
	C10-C11	1.3965	2.210	-22.658	0.16
			2.088	-17.405	0.19
			2.071	-20.500	0.21
	C11-C6	1.3982	2.121	-21.371	0.20
			2.087	-17.401	0.20
			2.067	-20.200	0.22
	C7-H7	1.0869	1.928	-23.980	0.05
			1.901	-20.441	0.03
			1.896	-23.200	0.02
	C8-H8	1.0856	1.753	-18.682	0.05
			1.896	-20.076	0.02
			1.894	-23.200	0.02
	C9-H9	1.0860	1.714	-20.759	0.02
			1.897	-20.321	0.02
			1.891	-23.100	0.02
	C10-H10	1.0882	1.860	-20.918	0.06
			1.899	-20.496	0.02
			1.889	-23.100	0.02

	C11-H11	1.0839	1.926	-23.574	0.05
			1.917	-20.900	0.03
			1.903	-23.400	0.02
20 K	C1-C2	1.4274	1.982	-18.499	0.21
			1.945	-14.782	0.21
			1.960	-18.160	0.20
	C2-C3	1.4068	2.088	-20.380	0.23
			2.028	-16.395	0.20
			2.039	-19.613	0.22
	C3-C4	1.4391	1.972	-18.349	0.17
			1.909	-14.842	0.15
			1.924	18.018	0.14
	C4-C5	1.3666	2.243	-23.613	0.26
			2.179	-18.977	0.24
			2.189	-22.515	0.26
	C4-H4	1.0839	1.857	-20.836	0.06
			1.913	-20.757	0.03
			1.917	-23.639	0.02
	C5-H5	1.0876	1.867	-21.924	0.06
			1.884	-20.680	0.03
			1.886	-23.037	0.02
	C2-C6	1.4979	1.705	-13.428	0.06
			1.729	-11.782	0.04
			1.735	-14.997	0.03
	C6-C7	1.4001	2.111	-20.808	0.28
			2.066	-16.956	0.18
			2.056	-20.046	0.21
	C7-C8	1.3941	2.074	-19.849	0.17
			2.095	-17.530	0.19

			2.084	-20.718	0.20
	C8-C9	1.3975	2.126	-20.683	0.16
			2.071	-16.930	0.19
			2.070	-20.509	0.20
	C9-C10	1.3965	2.128	-21.019	0.27
			2.070	-16.957	0.20
			2.069	-20.448	0.20
	C10-C11	1.3975	2.072	-20.369	0.22
			2.072	-17.014	0.19
			2.065	-20.336	0.21
	C11-C6	1.4005	2.106	-19.909	0.17
			2.069	-16.984	0.20
			2.057	-20.013	0.22
	C7-H7	1.0868	1.892	-21.802	0.04
			1.895	-20.337	0.03
			1.896	-23.211	0.02
	C8-H8	1.0855	1.874	-21.377	0.07
			1.894	-20.089	0.02
			1.894	-23.208	0.02
	C9-H9	1.0862	1.855	-21.617	0.04
			1.892	-20.262	0.02
			1.891	-23.133	0.02
	C10-H10	1.0865	1.864	-21.313	0.05
			1.894	-20.405	0.02
			1.889	-23.099	0.02
	C11-H11	1.0850	1.832	-21.273	0.09
			1.913	-20.828	0.03
			1.903	-23.386	0.02

Table S3 Integrated net atomic charges q(Ω) derived from the QTAIM analysis

	100 K				20 K			
Atom	Experiment	theory, multipole	theory, LCGTF	theory, Gaussian	experiment	theory, multipole	theory, LCGTF	theory, Gaussian
C1	-0.07	-0.03	0.00	-0.01	-0.05	-0.03	0.00	-0.01
C2	0.04	-0.02	0.01	-0.02	-0.02	-0.02	0.01	-0.02
C3	-0.01	-0.02	0.00	-0.01	-0.03	-0.02	0.00	-0.01
C4	-0.02	-0.08	0.02	-0.02	-0.03	-0.03	0.02	-0.02
C5	-0.02	-0.04	0.00	-0.02	-0.04	-0.02	0.00	-0.02
C6	-0.03	-0.02	-0.01	0.00	-0.03	-0.02	-0.01	0.00
C7	-0.07	-0.03	0.00	-0.01	-0.04	-0.02	0.00	-0.01
C8	-0.03	-0.04	0.01	-0.01	-0.02	-0.02	0.01	-0.01
C9	-0.04	-0.03	0.01	-0.01	-0.04	-0.02	0.01	-0.01
C10	-0.08	-0.01	0.01	-0.02	-0.03	-0.02	0.01	-0.01
C11	0.09	-0.03	0.01	-0.01	-0.02	-0.02	0.01	-0.01
H4	0.01	0.03	-0.01	0.04	0.05	0.03	-0.01	0.04
H5	-0.05	0.04	-0.01	0.02	0.04	0.04	-0.02	0.02
H7	0.05	0.04	-0.01	0.02	0.06	0.03	-0.01	0.02
H8	0.06	0.09	-0.01	0.01	0.04	0.03	-0.01	0.01
H9	0.09	0.08	0.00	0.02	0.05	0.03	0.00	0.02
H10	0.03	0.04	-0.02	0.02	0.05	0.04	-0.02	0.02
H11	0.05	0.03	-0.01	0.02	0.06	0.04	-0.01	0.02

/

Table S4 Lattice energy and intermolecular interaction energies of selected molecular dimers ($kJmol^{-1}$) in rubrene obtained from the PIXEL calculations using the ED from the MP2 method. The values reported in first and second lines correspond to the crystal geometry at 100 K and 20 K, respectively. Symmetry operations are listed in Table 1.

molecular dimers	Interaction Distance (Å)	Centroid-centroid distance (Å)	E_{es}	E_{pol}	E_{disp}	E_{rep}	E_{tot}
Lattice energy	-	-	–55.9	–34.8	–305.0	171.8	–223.9
	-	-	–57.4	–35.9	–309.7	178.2	–224.9
$C_\pi \cdots C_\pi$ stacking[a] (dimer I)	3.706(1)	7.160(2)	–8.1	–14.2	–110.5	64.0	–68.7
	3.694(1)	7.160(2)	–8.3	–14.4	–112.1	66.2	–68.6
C4⋯H5[b] (dimer II)	2.825(1)	7.953(3)	–15.8	–6.7	–62.1	36.5	–48.0
	2.817(1)	7.930(2)	–16.2	–7.0	–63.1	37.7	–48.5
H8⋯H8[d] (dimer III)	2.268(1)	13.875(4)	–7.0	–2.8	–24.2	15.2	–18.8
	2.264(1)	13.868(3)	–7.1	–2.9	–24.5	15.7	–18.8
H9⋯H9[e] (dimer IV)	2.667(1)	15.170(2)	–1.1	–0.4	–7.6	2.5	–5.5
	2.623(1)	15.152(2)	–1.2	–0.4	–7.8	2.7	–5.6

///
